# Supplementary material for: Effects of mycotoxin-producing fungi on the fitness and gut bacterial community of the soil springtail Folsomia candida
Source: Microbiol Spectr. 2024 Sep 27;12(11):e01035-24. doi: 10.1128/spectrum.01035-24 (PMC11537059; doi:10.1128/spectrum.01035-24)
Supplement: Supplemental material — Tables S1 to S7; Fig. S1 to S5. [file spectrum.01035-24-s0001.docx]

# Supplementary Information

**Effects of mycotoxin producing fungi on the fitness and gut microbial community of the soil springtail *Folsomia candida***

Yang Xu^a#^, Lingxiao Tang^a#^, Zhen Xie^b#^, Xingwei Duan^a^, Kaisha Wang^a^, Jialin Zhu^a^, Yangyang Huang^a^, Kailang Yang^a^, Lei Xu^b^*, Hong He^a^*

^a^Key Laboratory of National Forestry and Grassland Administration for Control of Forest Biological Disasters in Western China, College of Forestry, Northwest A&F University, Yangling, Shaanxi 712100, China.

^b^College of Life Sciences, Northwest A&F University, Yangling 712100, Shaanxi, China.

#These authors contributed equally in this work.

*Corresponding author at:

Hong He ([hehong@nwsuaf.edu.cn](mailto:hehong@nwsuaf.edu.cn)), Key Laboratory of National Forestry and Grassland Administration for Control of Forest Biological Disasters in Western China, College of Forestry, Northwest A&F University, Yangling, Shaanxi 712100, China;

Lei Xu ([xulei@nwafu.edu.cn](mailto:xulei@nwafu.edu.cn)), College of Life Sciences, Northwest A&F University, Yangling 712100, Shaanxi, China.

**Running title**: Fungi affect springtail’s fitness and gut bacteria.

# Supplementary Table 1. Primers were used in this study.

| **Primers** | **5’-3’ sequence** | **Amplicon size (bp)** | **Species** | **Reference** |
| --- | --- | --- | --- | --- |
| 341F | ACTCCTACGGGAGGCAGCAG | 465 | Bacteria | Dai et al., 2022 |
| 806R | GGACTACHVGGGTWTCTAAT |  |  |  |
| Ba 27F | AGAGTTTGATCCTGGCTCAG | 1465 | Bacteria | Lane et al., 1991 |
| Ba1492R | GGTTACCTTGTTACGACTT |  |  |  |
| VER1 | CTTCCTGCGATGTTTCTCC | 578 | *F. verticillioides* | (1) |
| VER1 | AATTGGCCATTGGTATTATATATCTA |  |  |  |
| Fg16N F | ACAGATGACAAGATTCAGGCACA | 280 | *F. graminearum* | (2) |
| Fg16N R | TTCTTTGACATCTGTTCAACCCA |  |  |  |
| AflR F | AGAGCCGCATGAGAGTATCC | 206 | *A. nidulans* | (3) |
| AflR R | CTCGCTTTCTCCTTCGCTTC |  |  |  |
| OCA F | ATACCACCGGGTCTAATGCA | 260 | *A. ochraceus* | (4) |
| OCA R | TGCCGACAGACCGAGTGGATT |  |  |  |

Dai, T., Wen, D., Bates, C.T. et al. Nutrient supply controls the linkage between species abundance and ecological interactions in marine bacterial communities. Nat Commun 13, 175 (2022). https://doi.org/10.1038/s41467-021-27857-6

Lane, D.J. (1991) 16S/23S rRNA sequencing. In: Nucleic Acid Techniques in Bacterial Systematics (Stackebrandt, E. and Goodfellow, M., Eds.), pp. 115–175. John Wiley and Sons, New York.

White, T.J., Bruns, T., Lee, S. and Taylor, J. (1990) Amplification and direct sequencing of fungal ribosomal RNA gene for phylogenetics. In PCR Protocols, A Guide to Methods and Applications (Innis, M. A., Gelfand, D. H., Sninsky, J.J. and White, T.J., Eds.), PP. 315-322. Academic Press, New York.

# Supplementary Table 2. Uniting the classification of cultured microorganisms and OTUs obtained from 16S rRNA (V3-V4) sequencing data.

| Isolated strain | Referred to OTU | Proportion of amplicon sequences of corresponding OTUs in fungi/bacterial community of each sample | | | | | | | | | | | | | | | | | |
| --- | --- | --- | --- | --- | --- | --- | --- | --- | --- | --- | --- | --- | --- | --- | --- | --- | --- | --- | --- |
|  |  | Yeast1 | Yeast2 | Yeast3 | Fv1 | Fv2 | Fv3 | Fg1 | Fg2 | Fg3 | An1 | An2 | An3 | Ao1 | Ao2 | Ao3 | Hunge1 | Hunge2 | Hunge3 |
| *Microbacterium* | OTU 1038, 10 | 3.93% | 0.24% | 12.44% | 0.07% | 0.04% | 0.16% | 0.33% | 0.63% | 0.49% | 3.14% | 6.14% | 0.18% | 12.72% | 10.86% | 2.76% | 5.55% | 9.86% | 4.96% |
| *Streptomyces* | OTU 334 | 0.06% | 0.01% | 0.07% | 0.02% | 0.03% | <0.005% | 0.04% | 0.08% | 0.08% | <0.005% | 0.04% | nd | nd | 0.19% | 0.05% | 0.03% | nd | nd |
| *Galactobacter* | nd |  |  |  |  |  |  |  |  |  |  |  |  |  |  |  |  |  |  |
| *Pimelobacter* | nd |  |  |  |  |  |  |  |  |  |  |  |  |  |  |  |  |  |  |
| *Arthrobacter* | OTU 447, 183, 22 | 2.33% | 0.07% | 2.79% | 0.02% | 0.04% | 0.02% | 0.03% | 0.04% | nd | 0.01% | nd | 0.01% | 0.09% | 0.41% | 0.08% | 0.24% | 0.23% | 0.28% |
| *Brachybacterium* | OTU 452 | nd | 0.01% | nd | 0.01% | nd | <0.005% | nd | md | nd | nd | nd | nd | nd | 0.04% | nd | nd | nd | nd |
| *Bacillus* | OTU 889, 773, 573, 200, 91, 86 | 0.13% | 0.04% | 0.26% | 0.02% | <0.005% | 0.03% | 0.08% | 0.16% | 0.08% | 0.03% | 0.01% | 0.06% | 0.06% | 1.21% | 0.01% | 0.02% | 0.07% | 0.25% |
| *Mammaliicoccus* | nd | Yeast1 | Yeast2 | Yeast3 | Fv1 | Fv2 | Fv3 | Fg1 | Fg2 | Fg3 | An1 | An2 | An3 | Ao1 | Ao2 | Ao3 | Hunger1 | Hunger2 | Hunger3 |
| *Staphylococcus* | OTU 67 27 | 0.38% | 0.20% | 0.46% | 0.02% | 0.08% | 0.17% | 0.17% | 0.47% | 0.27% | 0.46% | 0.29% | 0.13% | 0.47% | 1.28% | 0.22% | 0.63% | 0.33% | 3.72% |
| *Stenotrophomonas* | OTU 4 | 13.71% | 16.62% | 33.04% | 1.31% | 51.63% | 1.65% | 0.01% | 0.15% | 0.34% | nd | 0.06% | 0.12% | 0.08% | 4.24% | nd | <0.005% | <0.005% | <0.005% |
| *Paracoccus* | OTU5 | 0.26% | 0.06% | 0.10% | 42.92% | nd | 0.04% | nd | 0.17% | 0.09% | 0.02% | 6.29% | 0.02% | 2.90% | nd | 0.23% | 0.05% | 0.10% | 0.61% |
| *Brucella* | OTU3 | 14.40% | 55.67% | 17.00% | 0.30% | 1.11% | 0.33% | 21.32% | 9.07% | 14.11% | 2.54% | 8.08% | 0.06% | 0.42% | 0.83% | 2.50% | 2.23% | 0.99% | 0.21% |
| *Acinetobacter* | OTU 260, 899, 234, 734, 278, 73, 522, 355 | 0.30% | 0.09% | 0.29% | <0.005% | 0.22% | 0.08% | 0.13% | 0.24% | 0.21% | 0.32% | 0.12% | 0.20% | 0.16% | 0.86% | 0.41% | 0.13% | 0.21% | 0.27% |

# Supplementary Table 3. Common mycotoxin concentration in each mycotoxin producing fungi diet.

| Diet | Mycotoxin | Content |
| --- | --- | --- |
| Fv *(F. verticillioides)* | Fumonisin B1 | 1110585 μg/kg |
|  |  |  |
| Fg *(F. graminearum)* | Deoxynivalenol | 31602 μg/kg |
|  | Zearalenone | 34461 μg/kg |
|  |  |  |
| An *(A. nidulans)* | Sterigmatocystin | 25368 μg/kg |
|  |  |  |
| Ao *(A. ochraceus)* | Ochratoxin A | <0.5 μg/kg |

**Supplementary Table 4. Dominant gut bacterial phylum of *F. candida* with different dietary treatments.**

| Phylum | yeast | Fv | Fg | An | Ao | hunger |  |
| --- | --- | --- | --- | --- | --- | --- | --- |
| F1 *F. candida* | | | | | | | |
| Proteobacteria | 70.08% ± SE 13.14% | 72.13% ± SE 21.74% | 86.05% ± SE 1.67% | 72.80% ± SE 13.09% | 51.05% ± SE 10.45% | 43.94% ± SE 0.46% |  |
| Actinobacteria | 12.30% ± SE 5.53% | 21.83% ± SE 20.50% | 5.86% ± SE 1.28% | 10.19% ± SE 4.88% | 22.47% ± SE 4.86% | 43.14% ± SE 3.25% |  |
| Bacteroidetes | 14.36% ± SE 10.30% | 2.75% ± SE 1.34% | / | 8.64% ± SE 5.35% | / | / |  |
| Firmicutes | / | / | 2.89% ± SE 0.45% | / | / | 5.61% ± SE 1.83% |  |
| F2 *F. candida* | | | | | | | |
| Proteobacteria | 66.21% ± SE 9.11% | 23.89% ± SE 1.54% | 45.25% ± SE 8.09% | 33.02% ± SE 12.59% | 36.61% ± SE8.01% | 55.86% ± SE 7.30% |  |
| Firmicutes | 17.38% ± SE 7.43% | 63.17% ± SE 4.91% | 32.72% ± SE 7.76% | 45.20% ± SE 16.58% | 37.01% ± SE 8.31% | 21.67% ± SE 13.18% |  |
| Actinobacteria | 10.21% ± SE 2. 67% | 5.48% ± SE 3.53% | 8.66% ± SE 1.44% | 7.60% ± SE 1.79% | 10.11% ± SE 0.70% | 5.85% ± SE 3.14% |  |
| Bacteroidetes | 1.88% ± SE 0.34% | 5.19% ± SE 3.72% | 8.29% ± SE 2.44% | 5.24% ± SE 0.82% | 6.72% ± SE 3.33% | 15.32% ± SE 12.06% |  |

**Supplementary Table 5. Dominant gut bacterial genera of *F. candida* with different dietary treatments.**

| Genus | yeast | Fv | Fg | An | Ao | hunger |
| --- | --- | --- | --- | --- | --- | --- |
| F1 *F. candida* | | | | | | |
| *Brucella* | 29.03% ± SE 13.12% | / | 14.71% ± SE 3.70% | / | / | / |
| *Stenotrophomonas* | 21.12% ± SE 5.65% | 18.11% ± SE 16.90% | / | / | / | / |
| *Sphingobactertium* | 13.73% ± SE 10.05% | / | / | / | / | / |
| *Gordonia* | / | 18.97% ± SE 18.89% | / | / | 6.87%±SE 3.32% | 28.67% ± SE 3.86% |
| *Methyloversatilis* | / | / | 3.88% ± SE 2.18% | / | / | 11.23% ± SE 1.65% |
| *Microbacterium* | / | / | / | / | 8.72% ± SE 3.00% | 6.80% ± SE 1.44% |
| *Pseudomonas* | / | 35.32% ± SE 26.14% | / | / | / | / |
| *Gordonia* | / | / | / | 4.76% ± SE 2.45% | / | / |
| *Lysobacter* | / | / | 9.33% ± SE 4.58% | / | / | / |
| *Flavihumibacter* | / | / | / | 6.51% ± SE 4.69% | / | / |
| *Sphingopyxis* | / | / | / | 4.61% ± SE 2.31% | / | / |
| *Prevotella* | / | / | / | / | 5.24% ± SE 4.00% | / |
| F2 *F. candida* | | | | | | |
| *Staphylococcus* | 15.22% ± SE 7.21% | 42.38% ± SE 21.34% | 15.77% ± SE 4.43% | 40.67% ± SE 17.11% | 18.98% ± SE 14.88% | 17.49% ± SE 14.81% |
| *Stenotrophomonas* | 10.02% ± SE 7.67%) | / | / | / | / | 19.17% ± SE 10.75% |
| *Pseudomonas* | 7.31% ± SE 3.23% | / | / | / | / | / |
| *Escherichia* | / | 4.81% ± SE 4.76% | / | / | / | / |
| *Microbacterium* | / | 3.65% ± SE 3.51% | / | / | / | / |
| *Paracoccus* | / | / | 5.44% ± SE 1.75% | / | / | / |
| *Prevotella* | / | / | 4.50% ± SE 2.49% | / | / | / |
| *GPI* | / | / | / | 3.95% ± SE 3.42% | 4.31% ± SE 2.00% | / |
| *Sphingobacterium* | / | / | / | 2.85% ± SE 1.59% | / | 12.93% ± SE 12.33% |
| *Brucella* | / | / | / | / | 4.42% ± SE 2.20% | / |

## Supplementary Table 6. Comparison abundance of foodborne mycotoxin producing fungi in gut of *F1 F. candida* and their residue in *F2 F. candida*.

|  | Relative abundance | | | | | | | | | | | |
| --- | --- | --- | --- | --- | --- | --- | --- | --- | --- | --- | --- | --- |
|  | *F. verticillioides* | | | *F. graminearum* | | | *A. nidulans* | | | *A. ochraceus* | | |
|  | replicate 1 | replicate 2 | replicate 3 | replicate 1 | replicate 2 | replicate 3 | replicate 1 | replicate 2 | replicate 3 | replicate 1 | replicate 2 | replicate 3 |
| F1 *F. candida* | 0.090% | 13.71% | 0.00% | 1.30% | 1.13% | 0.00% | 15.72% | 12.78% | 1.76% | 48.94% | 88.16% | 70.56% |
| F2 *F. candida* | 0.27% | 0.40% | 0.007% | 0.00% | 0.51% | 0.05% | 0.54% | 0.28% | 0.15% | 0.59% | 0.03% | 0.11% |
| F1 *F. candida* /F2. *F. candida* ratio | 19.688100 | | | 4.285324 | | | 31.15827 | | | 284.1841 | | |
| t-test | *P* = 0.3555 | | | *P* = 0.2301 | | | *P* = 0.0833 | | | *P* = 0.0037, ** | | |

T-test analysis was employed to compare foodborne fungi in F1 *F. candida* and F2 *F. candida*. ***P*< 0.01

## Supplementary Table 7. Correlations between diet type, fitness parameter and gut microbiota change of *F. candida*.

| Variable | Diet type | F1 *F. candida* body length | F1 *F. candida* body width | F1 *F. candida* survival rate | No. of different gut bacteria | Different gut bacterial abundance |
| --- | --- | --- | --- | --- | --- | --- |
| Diet type | 1.000 | -.786^**^ | -.796^**^ | 0.060 | .949^**^ | .941^**^ |
| F1 *F. candida* body length | / | 1.000 | .918^**^ | 0.000 | -.725^**^ | -.717^**^ |
| F1 *F. candida* body width | / | / | 1.000 | 0.016 | -.759^**^ | -.736^**^ |
| F1 *F. candida* survival rate | / | / | / | 1.000 | 0.112 | -0.039 |
| No. of different bacteria | / | / | / | / | 1.000 | .962^**^ |
| Different gut bacterial abundance | / | / | / | / | / | 1.000 |

Diet type represents yeast and mycotoxin producing fungi *F. graminearum*, *F. verticillioides*, *A. nidulans* and *A. ochraceus* grow on rice medium. Spearman analysis was used to analyze the correlation between variables. ***P* < 0.01.

# Supplementary Figures


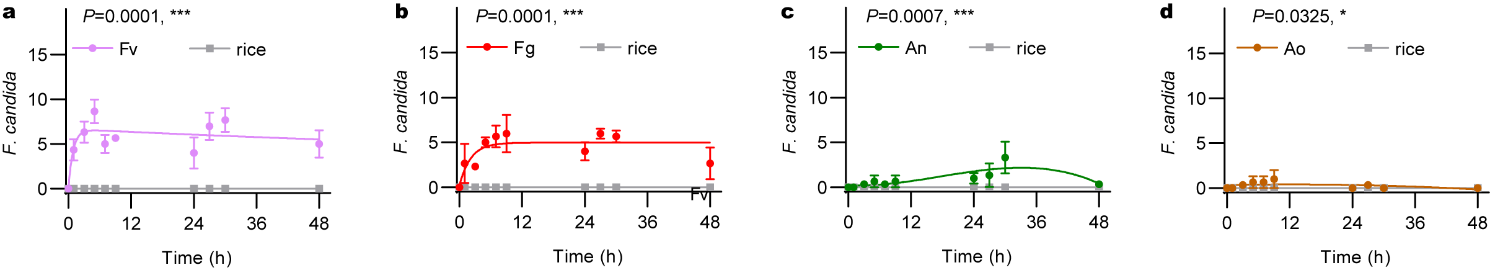


## Figure S1 Analysis of food preference between each mycotoxin producing fungi and rice medium. 20 *F. candida* were hungry for two days, then they were supplied a piece of two types of food a), *F. verticillioides* and rice; b): *F. graminearum* and rice; c): *A. nidulans* and rice; d): *A. ochraceus* and rice located at two sides of Petri dishes, the number of F. candida sit on each food at different time point were counted, this experiment were last for 48h, and performed under dark condition at room temperature. The comparison between the two groups was performed by the Mann-Whitney test.


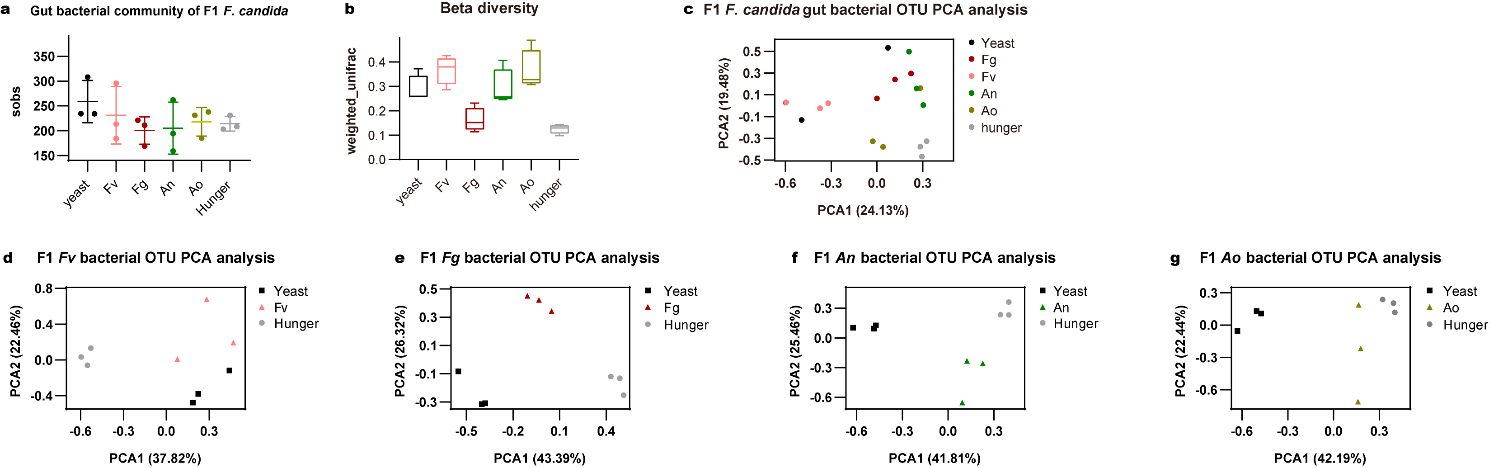


## Figure S2 Analysis of the bacterial 16S rRNA gene from F1 *F. candida* guts feeds on mycotoxin producing fungi.

**a**. Alpha diversity and the number of species observations (sobs) (abundance), **b**)**.** beta diversity (P = 0.024) based on operational taxonomic units (OTUs) derived from bacterial 16S rRNA gene from F1 *F. candida* guts feeds on mycotoxin-producing fungi. Principal component analysis (PCA) of pairwise distance among *F. candida* gut bacterial community feed on all diets **c**), *F. verticillioides* (Fv, **d**), *F. graminearum* (Fg, **e**), *A. nidulans* (An, **f**), *A. ochraceus* (Ao, **g**), Yeast and Hunger based on the Weighted Unifrac metrics (Adonis test).


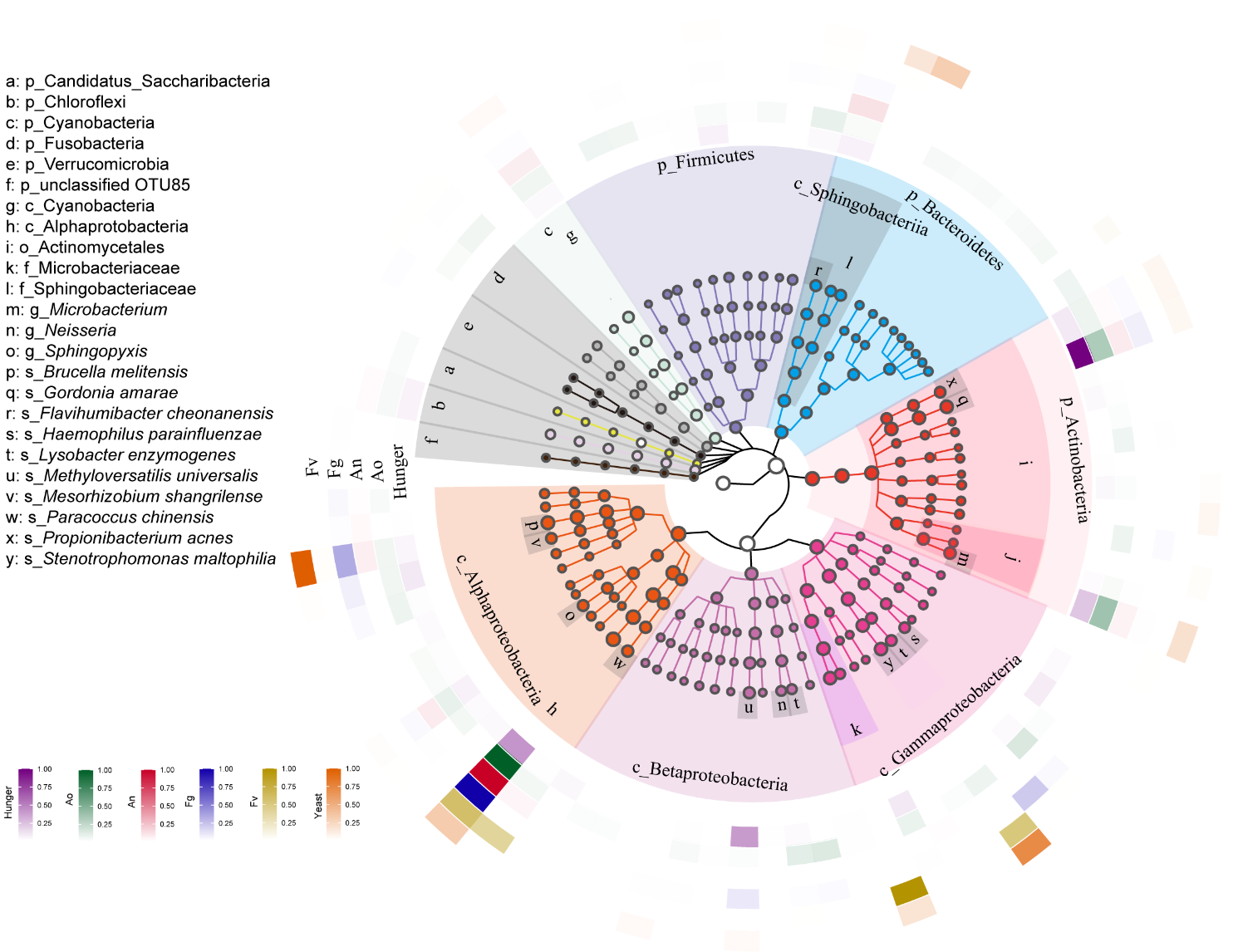


## Figure S3 Cladograms of bacterial communities in F1 *F. candida* guts feed on mycotoxin producing fungi.

The cladogram of enriched gut bacteria in F1 *F. candida* feeding on different diets was built using GraPhlAn software (https://huttenhower.sph.harvard.edu/graphlan). Cladogram results are plotted according to phylogeny, the size of nodes is proportional to the abundance of the taxon. The circles from inside to outside represent phylum, class, order, family, and genus, respectively. Different colors indicate distinct bacterial phylum. The peripheral circle is the thermodynamic diagram of abundance, each circle represents one group and corresponds to a color, and the color depth changes with the species richness. Cladograms show differences in taxa between the control (Yeast and Hunger) and mycotoxin producing fungi feeding groups.


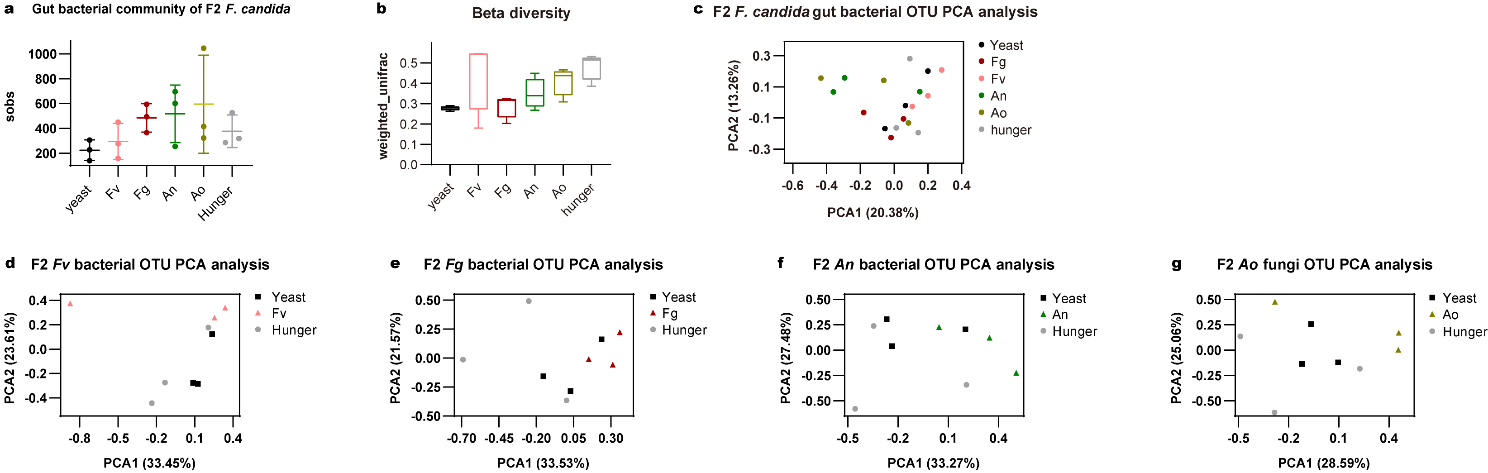


## Figure S4 Analysis of the bacterial 16S rRNA gene from F2 *F. candida* guts after feeding with normal diets.

**e**. Alpha diversity and the number of species observations (sobs) (abundance), **b**) beta diversity (p=0.255) based on operational taxonomic units (OTUs) derived from bacterial 16S rRNA gene from F2 *F. candida* guts after feeding with normal diets. Principal component analysis (PCA) of pairwise distance among gut bacterial community from F2 *F. candida* fed on yeast diets for six weeks that originated from the Yeast, all samples **c**), among *F. verticillioides* (Fv, **d**), *F. graminearum* (Fg, **e**), *A. ochraceus* (An, **f**), *A. nidulans* (Ao, **g**), yeast and Hunger F1 *F. candida* based on the Weighted Unifrac metrics (Adonis test).

**
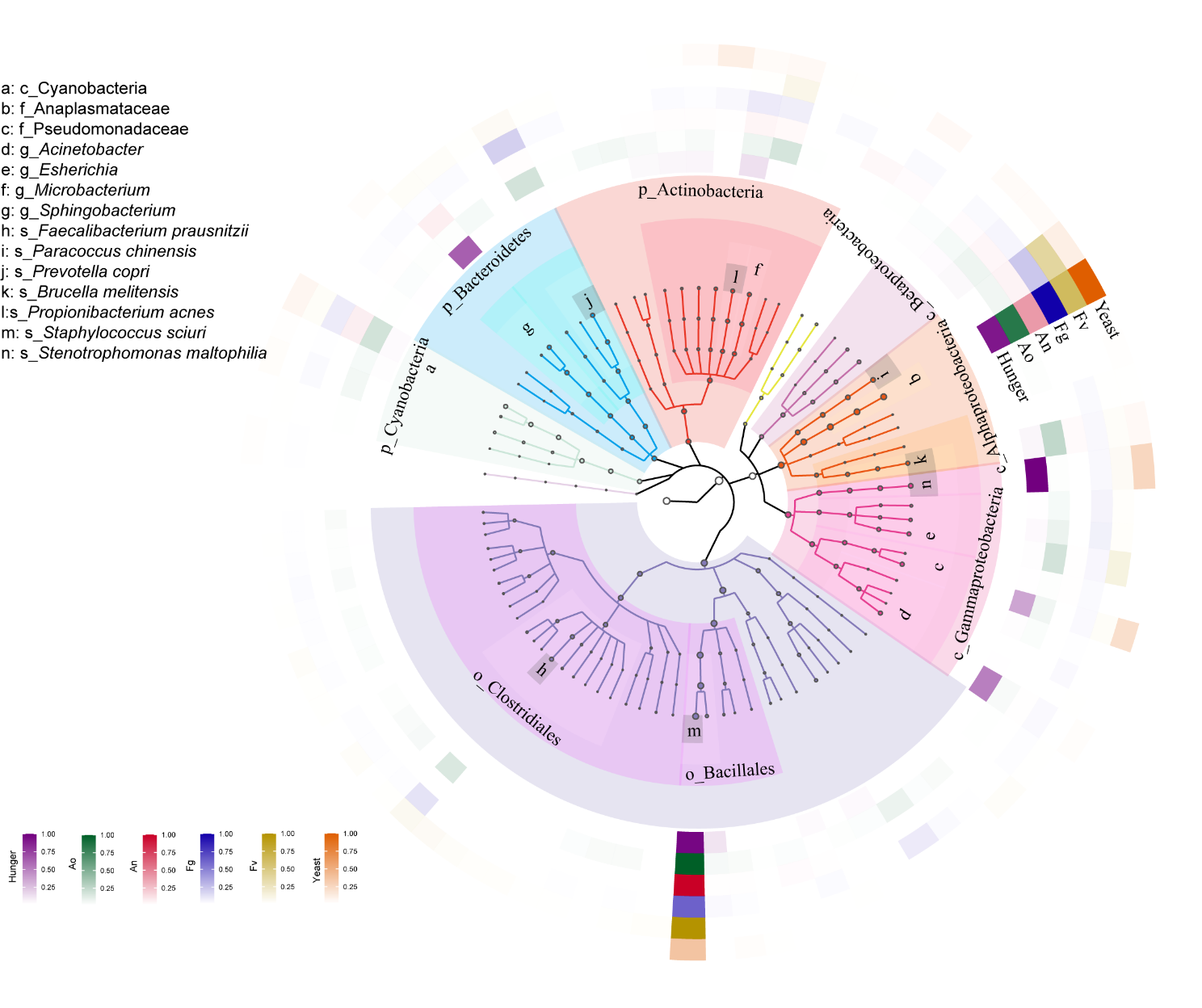
**

## Figure S5 Cladograms of bacterial communities in F2 *F. candida* guts after feeding with normal diets.

The Cladogram of enriched gut bacteria in F2 *F. candida* feeding on yeast was built using GraPhlAn software (https://huttenhower.sph.harvard.edu/graphlan). Cladogram results are plotted according to phylogeny, the size of nodes is proportional to the abundance of the taxon. The circles from inside to outside represent phylum, class, order, family, and genus, respectively. Different colors indicate distinct bacterial phylum. The peripheral circle is the thermodynamic diagram of abundance, each circle represents one group and corresponds to a color, and the color depth changes with the species richness.

# References

1. Harlapur SI, Ilger K, Salakinkop SR, Talekar SC, Kachapur RM, Balol G, Patil SB, Tippannavar PS. 2022. First report of *Fusarium verticillioides* causing Pokkah boeng disease on maize in India. Plant Dis doi:10.1094/PDIS-08-22-1764-PDN. <https://doi.org/10.1094/PDIS-08-22-1764-PDN>.

2. Nicholson P, Simpson DR, Weston G, Rezanoor HN, Lees AK, Parry DW, Joyce D. 1998. Detection and quantification of *Fusarium culmorum* and *Fusarium graminearum* in cereals using PCR assays. Physiol Mol Plant Pathol 53:17-37. <https://doi.org/10.1006/pmpp.1998.0170>.

3. Xu Y, Karlovsky P. 2018. Interactions Between Invertebrate and Mycotoxin-producing Fungi. Georg-August-Universität Göttingen.

4. Schmidt H, Bannier M, Vogel RF, Niessen L. 2004. Detection and quantification of *Aspergillus ochraceus* in green coffee by PCR. Lett Appl Microbiol 38:464-9. <https://doi.org/10.1111/j.1472-765X.2004.01524.x>.
